# Epigenetic therapy sensitizes anti–PD-1 refractory head and neck cancers to immunotherapy rechallenge

**DOI:** 10.1172/JCI181671

**Published:** 2025-03-17

**Authors:** Tingting Qin, Austin K. Mattox, Jean S. Campbell, Jong Chul Park, Kee-Young Shin, Shiting Li, Peter M. Sadow, William C. Faquin, Goran Micevic, Andrew J. Daniels, Robert Haddad, Christopher S. Garris, Mikael J. Pittet, Thorsten R. Mempel, Anne ONeill, Maureen A. Sartor, Sara I. Pai

**Affiliations:** 1 Department of Computational Medicine and Bioinformatics, University of Michigan, Ann Arbor, Michigan, USA.; 2 Department of Surgery, Division of Otolaryngology—Head and Neck Surgery, Yale University School of Medicine, New Haven, Connecticut, USA.; 3 Department of Surgery, and; 4 Department of Medicine, Massachusetts General Hospital, Boston, Massachusetts, USA.; 5 Department of Data Science, Dana-Farber Cancer Institute, Boston, Massachusetts, USA.; 6 Department of Pathology, Massachusetts General Hospital, Boston, Massachusetts, USA.; 7 Department of Dermatology, and; 8 Department of Immunobiology, Yale University School of Medicine, New Haven, Connecticut, USA.; 9 Department of Medical Oncology, Center for Head and Neck Oncology, Dana-Farber Cancer Institute, Boston, Massachusetts, USA.; 10 Center for Systems Biology, Massachusetts General Hospital, Boston, Massachusetts, USA.; 11 University of Geneva, Geneva, Switzerland.; 12 AGORA Cancer Center and Swiss Cancer Center Leman, Lausanne, Switzerland.; 13 Ludwig Institute for Cancer Research, Lausanne, Switzerland.; 14 Center for Immunology and Inflammatory Diseases, Division of Rheumatology, Allergy and Immunology, Massachusetts General Hospital and Harvard Medical School, Boston, Massachusetts, USA.; 15 Cancer Immunology Program, Dana-Farber Harvard Cancer Center, Boston, Massachusetts, USA.

**Keywords:** Immunology, Oncology, Cancer immunotherapy, Epigenetics, Head and neck cancer

## Abstract

**BACKGROUND:**

Immune checkpoint blockade (ICB) is an effective treatment in a subset of patients diagnosed with head and neck squamous cell carcinoma (HNSCC); however, the majority of patients are refractory.

**METHODS:**

In a nonrandomized, open-label Phase 1b clinical trial, participants with recurrent and/or metastatic (R/M) HNSCC were treated with low-dose 5-azacytidine (5-aza) daily for either 5 or 10 days in combination with durvalumab and tremelimumab after progression on ICB. The primary objective was to assess the biologically effective dose of 5-aza as determined by molecular changes in paired baseline and on-treatment tumor biopsies; the secondary objective was safety.

**RESULTS:**

Thirty-eight percent (3 of 8) of participants with evaluable paired tissue samples had a greater-than 2-fold increase from baseline in IFN-γ signature and *CD274* (programmed cell death protein 1 ligand, PD-L1) expression within the tumor microenvironment (TME), which was associated with increased CD8^+^ T cell infiltration and decreased infiltration of CD4^+^ T regulatory cells. The mean neutrophil-to-lymphocyte ratio (NLR) decreased by greater than 50%, from 14.2 (SD 22.6) to 6.9 (SD 5.2). Median overall survival (OS) was 16.3 months (95% CI 1.9, NA), 2-year OS rate was 24.7% (95% CI: 4.5%, 53.2%), and 58% (7 of 12) of treated participants demonstrated prolonged OS of greater than 12 months.

**CONCLUSION:**

Our findings suggest that low-dose 5-aza can reprogram systemic host immune responses and the local TME to increase IFN-γ and PD-L1 expression. The increased expression of these established biomarkers correlated with prolonged OS upon ICB rechallenge.

**TRIAL REGISTRATION:**

ClinicalTrials.gov NCT03019003.

**FUNDING:**

NIH/NCI P01 CA240239.

## Abstract 2

5’aza therapy increased IFN-ɣ signature and PD-L1 expression and decreased NLR, which correlated with prolonged OS, in R/M HNSCC patients previously refractory to ICB.

## Introduction

The overall survival (OS) of patients diagnosed with recurrent and/or metastatic (R/M) head and neck squamous cell carcinoma (HNSCC) can be improved by targeting the PD-1:PD-L1 axis (1–4). However, improved OS is achieved in only 18%–20% of patients with R/M HNSCC (2). Increasing evidence highlights 2 main barriers to achieving clinical responses with immunotherapy in cancer patients: (a) the tumor’s overall poor antigenicity, which limits the generation of antitumor immunity, and (b) innate and adaptive immune suppressive mechanisms that result in immune tolerance. The overall poor antigenicity of HNSCCs, which can be attributed in part to the downregulation of the HLA class I antigen processing machinery (APM) components and immunogenic antigens through epigenetic silencing, can result in a “cold” immunophenotype (5). In this clinical study, we hypothesized that epigenetic reprogramming through the administration of a low-dose DNA methyltransferase inhibitor, 5-azacytidine (5-aza), can improve tumor antigenicity through epigenetic modulation of immune suppressive cells and the reexpression of epigenetically silenced HLA class I APM components and/or immunogenic tumor antigens (TAs) to improve clinical outcomes upon rechallenge with immune checkpoint blockade (ICB). A Phase 1b clinical trial was performed to assess the efficacy of low-dose 5-aza on reprogramming the tumor and immune cells within the tumor microenvironment (TME) in patients diagnosed with R/M HNSCC who were refractory to anti-PD-1 therapy.

## Results

### Participants

Between March 31, 2017, and November 4, 2020, 13 participants were enrolled. The trial was conducted during the COVID-19 pandemic, and, due to the hospital constraints surrounding the pandemic, the trial terminated before completing enrollment. This analysis focuses on the participants (*n* = 12) who received 5-aza subcutaneously. The median age was 62.1 years (range: 50.2–75.0), 92% (11 of 12) were male and 8% (1 of 12) were female, and 50% (6 of 12) had oropharyngeal primaries, followed by 25% (3 of 12) oral cavity, and 17% (2 of 12) larynx (Table 1). Of the participants with oropharyngeal cancer, 83% (5 of 6) were HPV16-mediated, as determined by HPV-16 RNA ISH testing (Table 2).

The primary therapy at the time of the initial diagnosis of HNSCC was the following: 58% (7 of 12) were treated with concurrent chemoradiation therapy; 25% (3 of 12) received induction chemotherapy followed by concurrent chemoradiation therapy; and 17% (2 of 12) underwent surgical resection followed by adjuvant chemoradiation therapy. Seventy-five percent (9 of 12) of our participants developed metastatic disease. Three patients (3 of 12, 25%) had persistent locoregional disease after treatment with concurrent chemoradiation therapy at the time of the initial cancer diagnosis. The overall mean disease-free interval (DFI) for those participants who developed metastatic disease was 8.5 months (Supplemental Table 1; supplemental material available online with this article; https://doi.org/10.1172/JCI181671DS1). DFI was calculated as the time from date of the last treatment that was administered at the time of initial cancer diagnosis to the date of diagnosis of metastatic disease.

As part of the trial inclusion criteria, all participants received and progressed on immunotherapy prior to enrolling in this trial: 50% (6 of 12) progressed on nivolumab, 42% (5 of 12) progressed on pembrolizumab, and 8% (1 of 12) progressed on durvalumab. A total of 56% (5 of 9) had prior radiation therapy for the treatment of metastatic disease and 33% (3 of 9) had received prior chemotherapy for metastatic disease. Eleven percent (1 of 9) had undergone prior metastasectomy to treat distant metastatic disease. Subsequent treatments received after the completion of this trial are outlined in Supplemental Table 2.

### Treatment and safety

**Figure 1:**
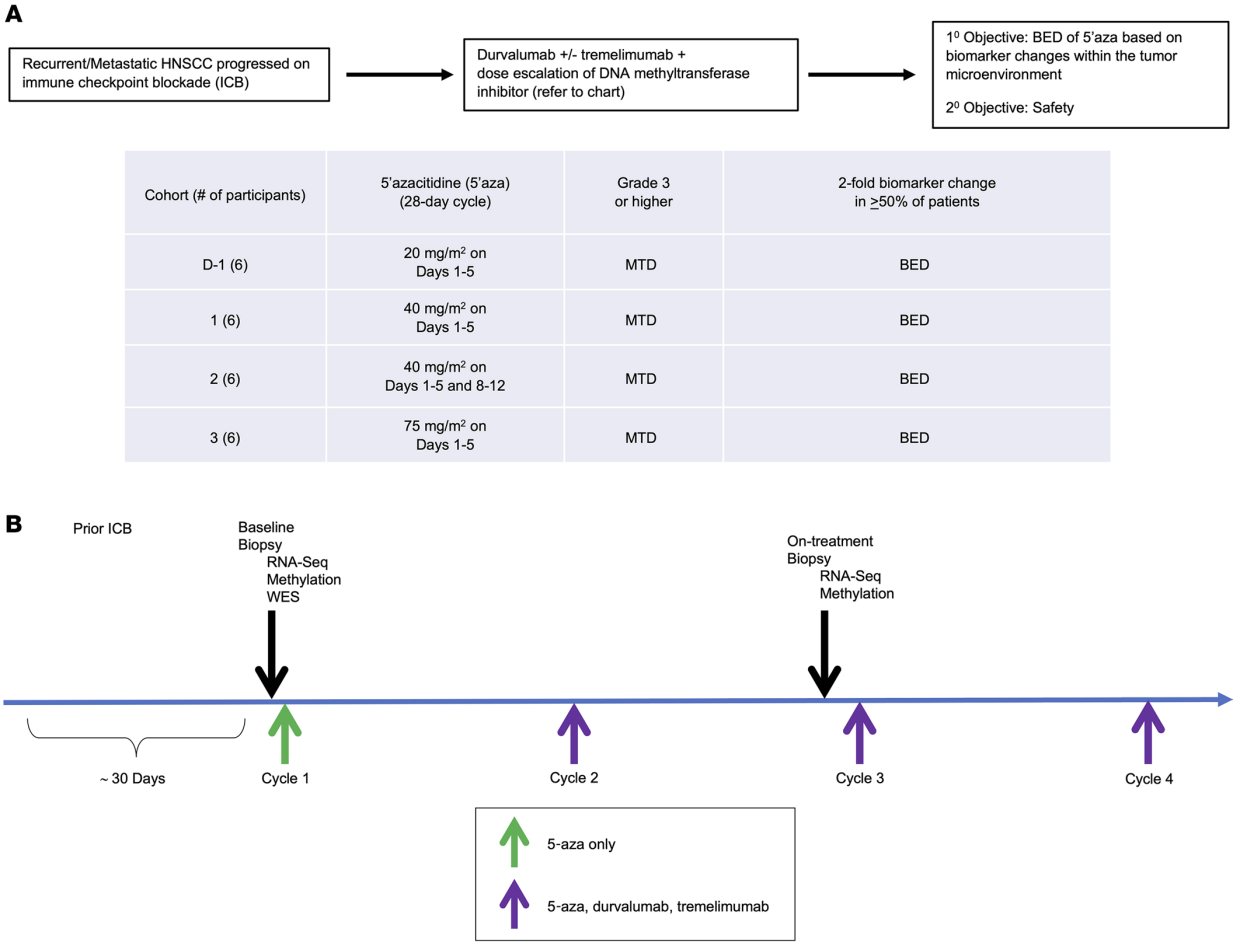
Clinical trial schema and multi-omics datasets included in the study. (**A**) Participants with locoregional or metastatic head and neck cancer that progressed on prior ICB were enrolled into the clinical trial (www.clinicaltrials.gov; NCT3019003). Participants were treated with escalating doses of a DNA methyltransferase inhibitor, 5-azacytidine (5-aza), and fixed doses of durvalumab (α-PD-L1) and tremelimumab (α-CTLA-4). The primary objective was determining the biologically effective dose (BED) of 5-aza. The secondary outcome was assessing the safety of the combination therapy. (**B**) Tissue specimens were collected (black arrows) prior to 5-aza treatment (green arrow) and after combination therapy with durvalumab and tremelimumab (purple arrows), which were given at the same time as the second dose of 5-aza (on-treatment).

The study design and participating patients are outlined in Figure 1. Low-dose 5-aza (40 mg/m^2^) was administered daily for either 5 or 10 days to assess whether the duration of treatment with 5-aza resulted in differences in toxicity and/or in epigenetic reprogramming of the TME. Seven participants received 5-aza for 5 days per cycle (dose 1), and 5 participants received 5-aza for 10 days per cycle (dose 2). There was 1 grade 4 treatment-related neutrophil count decrease and 1 grade 3 febrile neutropenia in participants treated with 10 days of 5-aza (Table 3). Treatment-related toxicities in the 5-day cohort included 1 grade 2 diarrhea, as well as grade 1 toxicities such as nausea, fatigue, arthralgia, and pruritus. There was a slight increase in median time-on-treatment in the 5-day cohort compared with the 10-day cohort (median 3.7 months [range 1.1–5.7 months] versus 2.8 months [0.1–3.9 months], respectively), suggesting that low-dose 5-aza of 40 mg/m^2^ administered for 5 days per cycle may be better tolerated than 10 days per cycle.

### Clinical outcome

**Figure 2:**
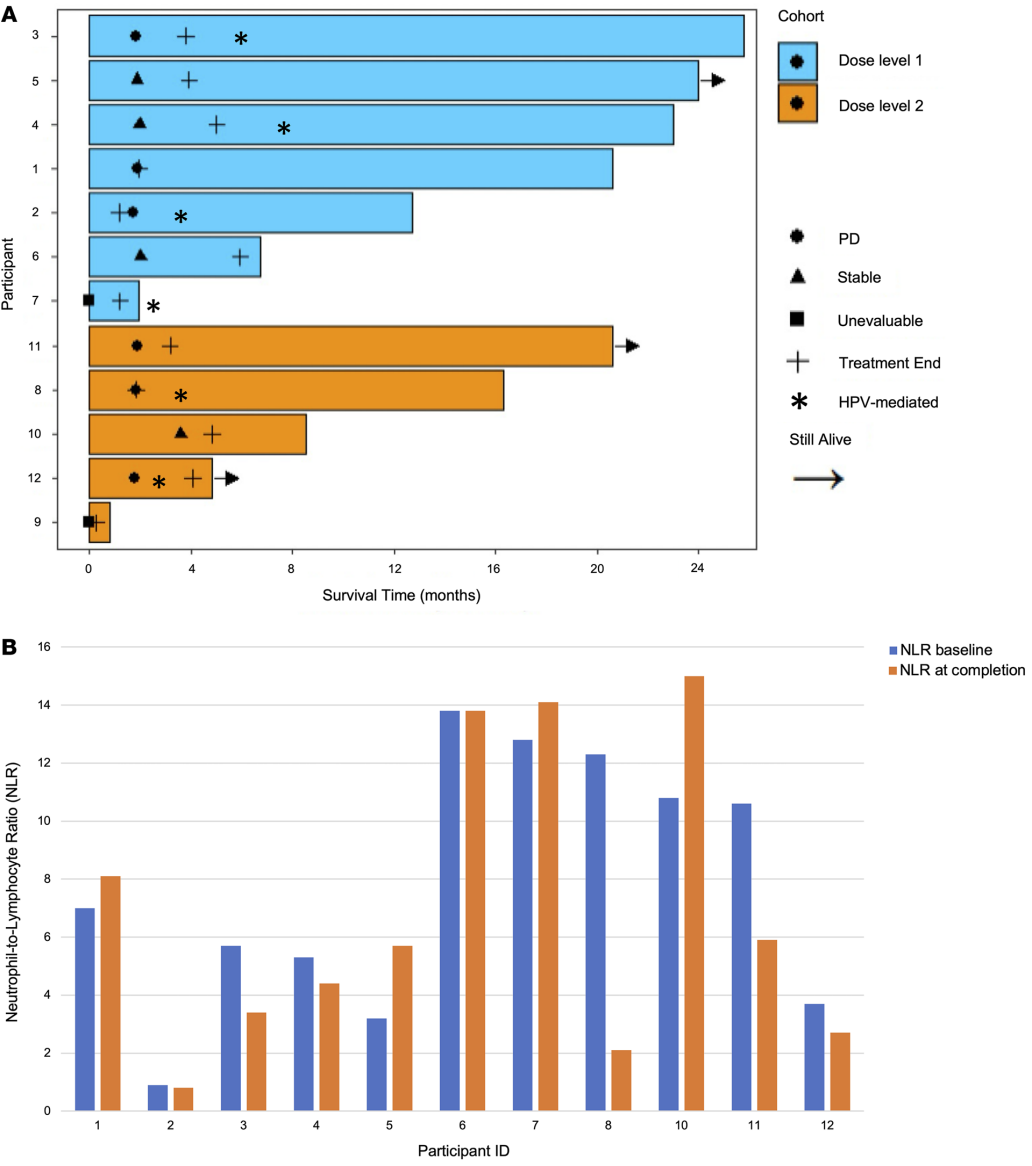
Swimmer plot of overall survival. (**A**) Swimmer plot demonstrates each participant’s (deidentified IDs in the y-axis) response to treatment in months (x-axis). Blue and orange bars denote Dose level 1 (5-days of 5-aza treatment) and Dose level 2 (10-days of 5-aza treatment), respectively. Participants with HPV-mediated disease are labeled with an asterisk (*). (**B**) The neutrophil-to-lymphocyte ratio at baseline (blue bars) and upon completion of the study (orange bars) is plotted for each participant.

Clinical responses were assessed by RECIST 1.1 at 8 weeks. Thirty-three percent (4 of 12) experienced stable disease as best overall response (Supplemental Table 3). The 3-month progression free survival (PFS) was 42.9% (95% CI: 9.8%, 73.4%) for dose level 1, 20% (95% CI: 0.8%, 58.2%) for dose level 2, and 33.3% (95% CI: 10.3%, 58.8%) overall (Supplemental Table 4). Of particular interest, at a median follow up of 20.6 months (range: 4.9–24.0 months), median OS was 16.3 months (95% CI 1.9, NA), 1-year OS rate was 64.8% (95% CI: 31.0%, 85.2%) and 2-year OS rate was 24.7% (95% CI: 4.5%, 53.2%) (Figure 2A). Fifty-eight percent (7 of 12) of treated participants demonstrated prolonged OS of greater than 12 months. Three participants (participants 5, 11, and 12; 1 HPV-mediated and 2 HPV-nonmediated HNSCCs, respectively) were still alive at time of final analysis. Due to the COVID-19 pandemic and associated hospital restrictions, an on-treatment research biopsy was unable to be obtained for participant 12. However, participants 5 and 11 had relevant changes in the TME as assessed by biomarker analyses.

### Biomarker outcomes

#### Neutrophil-to-Lymphocyte ratio.

In cancer patients, a pretreatment neutrophil-to-lymphocyte ratio (NLR) greater-than 10 is associated with poorer survival outcomes, with a reported median OS of 3.8 months (6). Several studies have reported on the association between NLR and clinical efficacy in cancer patients treated with immunotherapy. A NLR above 10 is also associated with a worse prognosis in patients receiving immunotherapy. In this trial, the mean NLR of the overall cohort (*n* = 12) at the time of administration of the first cycle of 5-aza was 14.2 (SD 22.6). At the last cycle of study treatment, the mean NLR of the overall cohort (*n* = 11) decreased by greater than 50% to 6.9 (SD 5.2). Participant 9 had only a baseline NLR because this participant received only 1 cycle of study drug before experiencing disease progression (NLR at baseline was 85). Figure 2B depicts the individual participants with matched NLR at baseline and at the time of last cycle of study therapy. Seventy-five percent (3 of 4) of participants in dose level 2 (participants 8, 10, 11) had an NLR of greater than 10 at baseline. Of these participants, 67% (2 of 3, participants 8 and 11) had a reduction in NLR after 5-aza treatment. The change in NLR in the overall cohort and individual participants suggest that 5-aza treatment was modulating systemic host immune responses in a favorable clinical direction (Supplemental Table 5). The paired tissue sample analyses confirm local immune reprogramming within the TME after 5-aza therapy.

### Tissue sample availability

A schematic of the clinical trial procedures is shown in Figure 1B. Due to the COVID-19 pandemic, there were challenges in obtaining the paired on-treatment tissue biopsies required for this clinical trial. Thus, of the total 12 participants treated, 5 participants (participants 2, 3, 4, 5, and 6) treated with 5-aza for 5 days and 3 participants (participants 8, 10, and 11) treated with 5-aza for 10 days had paired baseline and on-treatment tissue biopsies and are included in the correlative biomarker analyses (Supplemental Table 6).

### Low-dose 5-aza is linked to differential changes in global methylation based on the baseline epigenetic signature

Changes in global methylation within the TME after 5-aza treatment were assessed in all participants using the Illumina HumanMethylation EPIC BeadChip (Supplemental Figure 1A). Seventy-five percent of participants (6 of 8) experienced a decrease in global methylation after 5-aza treatment. However, the extent to which global methylation decreased varied between participants based on their baseline epigenetic signature. No statistically significant difference in the frequency of methylation changes within the promoters or CpG sites was detected between the 5-day and 10-day 5-aza treatment groups using a cut off of greater-than 25% methylation decrease (Supplemental Figure 1, B–F). Neither was there a strong methylation effect across differentially methylated regions (DMRs) among participants. For example, only 10 DMRs (adjusted *P* < 0.05) were identified with the 5-day 5-aza treatment and 11 DMRs with the 10-day treatment. Therefore, treatment with low-dose 5-aza for 5 days versus 10 days resulted in a similar global methylation effect, although the specific methylation changes varied among participants based on their individual baseline epigenetic fingerprint.

### Low-dose 5-aza can upregulate IFN-γ signature and CD274 (PD-L1) expression within the TME

**Figure 3:**
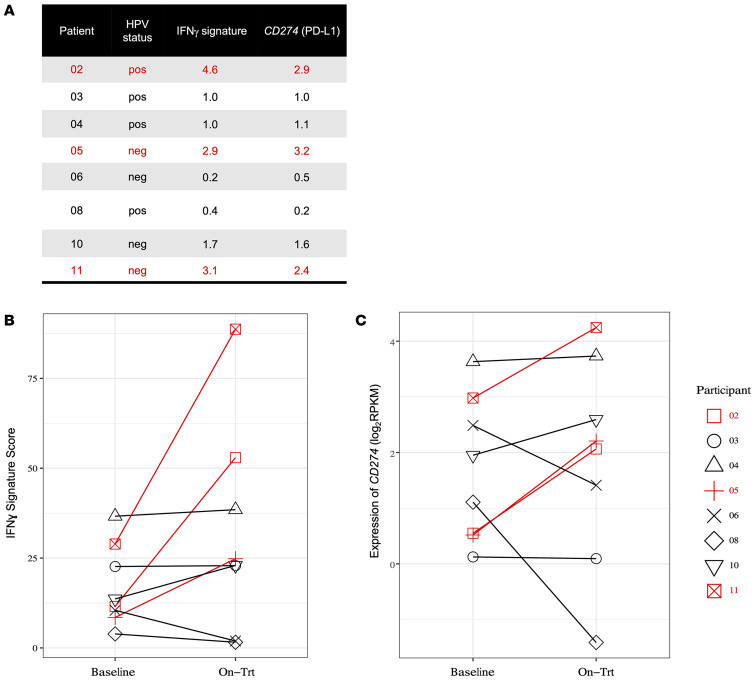
The IFN-γ signature and *CD274* expression increased by greater than 2-fold with 5-azacytidine in a subset of patients. (**A**) Summary of plotted data. (**B**) IFN-γ signature score. (**C**) Expression of *CD274* (PD-L1). Participants 2, 5, and 11 (red) showed significant increase in FC compared with others (black) (*P* = 0.036 for IFN-γ signature score and *P* = 0.036 for *CD274* expression).

To assess whether low-dose 5-aza treatment can improve tumor immunogenicity, we evaluated changes in the IFN-γ signature score based on 6 genes known to be involved in IFN-γ pathway signaling (i.e., *IDO1, CXCL10, CXCL9, HLA-DRA, STAT1,* and *IFNG)* (7) and evaluated the change in expression of *CD274* between paired baseline and on-treatment biopsies for individual participants. Three participants [participants 2 and 5 (both 5-day treatment) and participant 11 (10-day treatment)] demonstrated a greater-than 2-fold increase in the IFN-γ signature score, which was significantly higher than the fold change in other participants (average FC: 3.5 versus 0.8, *P* = 0.036) (Figure 3, A and B). In addition, the expression of *CD274*, which is the single best clinically proven biomarker of response to ICB (8), increased more than 2-fold in participants 2, 5, and 11, which was significantly higher than others (average FC: 2.8 versus 0.9, *P* = 0.036) (Figure 3, A and C). Low dose 5-aza (40 mg/m^2^) administered for 5 days was identified as the biologically effective dose (BED), and participants 2, 5, and 11 were considered molecular responders in the study.

We next determined whether the increases in IFN-γ signature and *CD274* expression were either a result of the direct or indirect hypomethylation of these genes by 5-aza. On-treatment hypomethylation of the promoter region of *CD274* was observed only in participants 3, 4, and 6, but there was no correlation between hypomethylation and expression changes of the IFN-γ signature genes and *CD274* in the available paired tissue samples (data not shown). Thus, these 3 participants (participants 2, 5, and 11) had substantial increases in biomarker expression within the TME, and 2 of these 3 patients (participants 5 and 11) were alive at 24.0 and 20.6 months after trial registration, respectively, at the time of final analysis. Ongoing post-study surveillance of 1 participant (participant 11) has shown no evidence of disease for over 3 years. Participant 2 began hospice shortly after stopping the trial and all treatment was ceased. Based on these molecular and clinical results, we investigated possible mechanism(s) for the observed increased proinflammatory TME with the administration of low dose 5-aza.

### Cell type deconvolution reveals increased infiltration of CD8+ T cells and decreased CD4+ T regulatory cells

**Figure 4:**
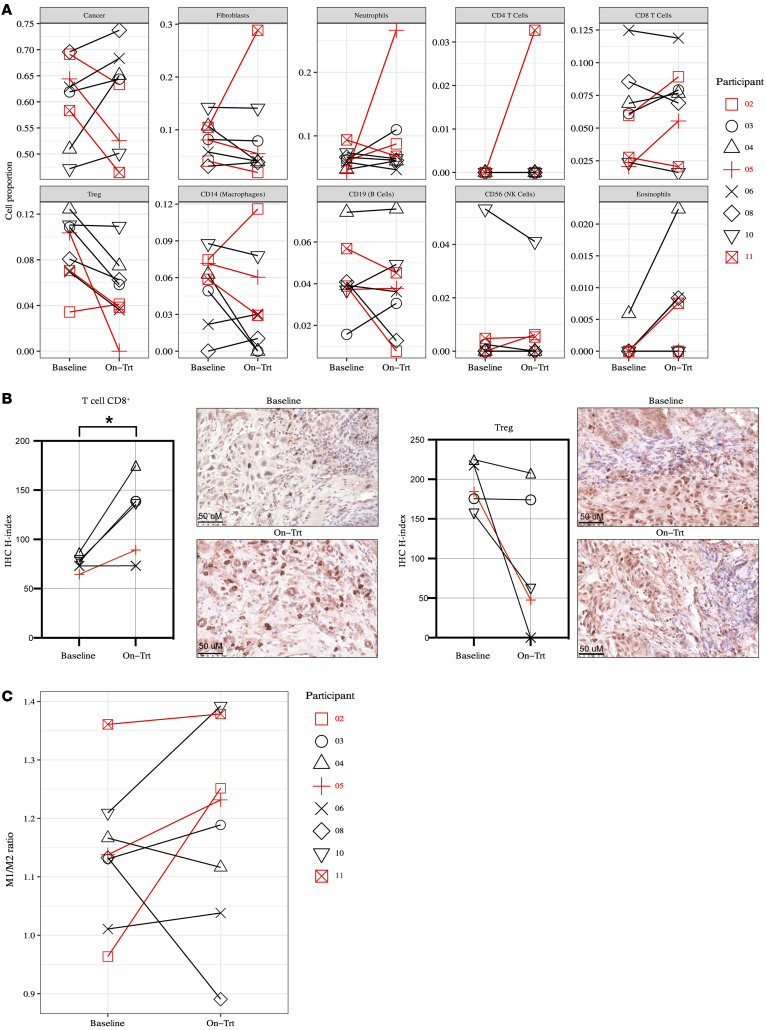
Cellular deconvolution indicates a decreased proportion of cancer cells in molecular responders, accompanied by an increased frequency of immune effector cells and decreased frequency of T_regs_. (**A**) Methylation-based deconvolution shows that the proportion of cancer cells decreased in participants 2, 5, and 11 (responders, red) compared with nonresponders (black), which was associated with an increase infiltration of CD8^+^ T cells (participants 2 and 5) and fibroblasts (participant 11). The proportion of T_regs_ decreased with 5-aza based on DNA methylation deconvolution. (**B**) Membrane CD8 and nuclear FOXP3 expression was quantified and analyzed in the baseline and on-treatment tissue sections. *P < 0.05, paired *t* test. (**C**) The expression ratio of M1-polarized macrophages versus M2 macrophages between baseline and on-treatment samples also increased in 75% of participants. Scale bars: 50 μm.

To investigate the inflammatory changes induced within the TME, we characterized the tumor-infiltrating immune and nonimmune cells in each participant using cell type deconvolution with the DNA methylation dataset. We estimated the proportions of 10 cell types, including cancer cells and 9 noncancer cell types. The proportions of CD4^+^ T regulatory cells (T_regs_) decreased by an average of approximately 35% across all 8 participants with evaluable matched pre- and on-treatment biopsies, suggesting a strong influence of epigenetic regulation of the *FOXP3* locus with low dose 5-aza treatment (9, 10) (Figure 4A). Correspondingly, infiltrating CD8^+^ T cells were increased in 50% (4 of 8) of participants with paired biopsy samples after study drug treatment: participant 2 (approximately 50% increase relative to baseline), participant 3 (approximately 30% increase relative to baseline), participant 4 (approximately 11% increase relative to baseline), and participant 5 (approximately 169% increase relative to baseline) (Figure 4A). To confirm these transcriptional changes, we performed IHC for CD8^+^ and FOXP3^+^ T cells on the paired pre- and on-treatment biopsy samples. We demonstrated that there was an increase in CD8^+^ T cells and decrease in FOXP3^+^ T cells after 5-aza treatment (Figure 4B).

As a high CD32, CD64, CD68, CD80, CD86, and IFN-γ–expressing M1 to CD163 and CD204 M2 macrophage polarization often correlates with a better prognosis in patients with cancer (11), we also calculated M1/M2 ratios based on the expression of 188 genes associated with M1 and 159 genes associated with M2 gene signatures (12) and evaluated the values in individual participants at baseline and on treatment. The expression ratio of M1-polarized macrophages versus M2-polarized macrophages between baseline and on-treatment samples increased in 75% (6 of 8) of participants (Figure 4C). Participant 2 demonstrated the highest fold change in M1/M2 ratio (FC = 1.3). Overall, these findings demonstrate that 2 participants (participants 2 and 5) had a greater than 50% increase in infiltrating cytotoxic CD8^+^ T cells, up to 10% decrease in infiltrating immune suppressive CD4^+^ T_regs_ cells, and an increase in the M1/M2 ratio, which correlated with the observed 2-fold increase in an IFN-γ signature and *CD274* expression within the TME (Figure 3). These changes translated into a reduction in the proportion of cancer cells in the on-treatment biopsies (approximately 8% in participant 2 and approximately 18% in participant 5 relative to baseline) (Figure 4A) and improved OS (12.7 months and 24.0 months, respectively).

### Enhanced expression of genes related to antigen presentation, processing, and antigenicity

**Figure 5:**
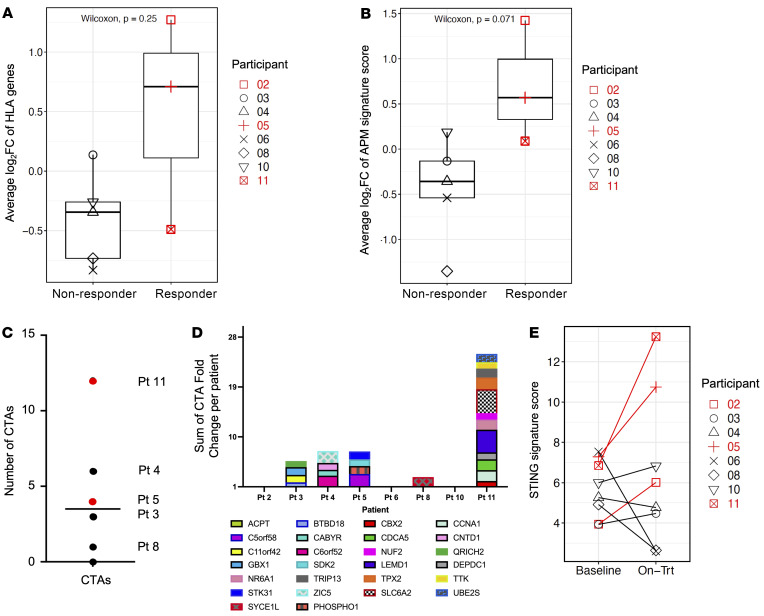
Fold change expression of genes relevant to tumor antigenicity after 5-aza treatment. (**A**) Participants 5 and 11 had increased expression of HLA and (**B**) APM compared with participants that did not respond to 5-aza treatment. (**C** and **D**) Participant 11 had the greatest number and diversity of cancer testis antigen (CTA), demonstrating at least a 2-fold-change in expression, while participants 2, 6, and 10 did not express any CTAs. (**E**) Participants 2, 5, and 11 had increased expression of STING pathway genes compared with participants that did not respond to 5-aza treatment.

We also performed whole exome sequencing (WES) to determine whether mutations in HLA or APM genes may be the reason that HNSCC participants are resistant to ICB. Only 1 participant (participant 3) with HPV-mediated HNSCC had 2 frameshift mutations identified in HLA-A (HLA-A* 68010102) (Supplemental Table 7), which may explain the lack of clinical and molecular response in this participant. Although B2M truncating mutations are more frequent in HPV-mediated HNSCCs (13), we did not observe any deleterious mutations in the B2M gene in any of the trial participants. Thus, HLA and APM mutations were not deemed a major mechanism for the progression on ICB in our study participants. Since HLA mutations were unlikely to be a primary reason for progression on ICB, we next assessed whether epigenetic silencing of HLA and APM genes, a known mechanism for tumor immune evasion in HNSCC (14, 15), could be reversed with low-dose 5-aza treatment. Gene set enrichment (GSE) of cytobands testing between on-treatment versus baseline samples revealed that cytoband 6p21.3 was significantly upregulated (FDR = 6.8710^–13^) among the 5-day treated participants. This cytoband contains a cluster of HLA complex genes that encodes cell-surface proteins responsible for presenting antigens on the cell surface. Thus, we hypothesized that 5-aza treatment may also impact HLA Class I APM expression. Participants 2 and 5 demonstrated an overall upregulation in fold-change of HLA gene expression by 1.6 and 2.5, respectively (Figure 5A). All 18 HLA genes at cytoband 6p21.3 were upregulated in participant 2, and 17 out of the 18 were upregulated in participant 5 (Supplemental Figure 2). Furthermore, increased expression of a broader set of genes related to antigen presentation and processing machinery was observed in the same 2 participants (Figure 5B and Supplemental Table 8).

In the setting of increased HLA and APM expression, we also explored changes in the expression of neoantigens and cancer testis antigens (CTAs), which could potentially expand the presented TA repertoire to induce a robust antitumor cell-mediated immune response (16, 17). We investigated the expression and methylation changes of 1,019 CTA genes (Supplemental Table 9). In total, 23 CTA genes were found to be upregulated (greater-than 2-fold change) in the TME by 5-aza treatment in all 8 participants with paired baseline and on-treatment biopsies, of which 17% (4 of 23) were increased in participant 5 and 52% (12 of 23) in participant 11 (Figure 5, C and D). The increased CTA repertoire after low-dose 5-aza could be responsible for the observed changes in the IFN-γ signature and *CD274* expression in participant 11, which was accompanied by a 20% reduction in the proportion of cancer cells in the TME after 5-aza treatment.

The Stimulator of Interferon Genes (STING) pathway plays a critical role in the innate immune response to viral infections (18, 19), and it has been reported that hypomethylating agents can induce STING activation mediated by the DNA sensor cyclic GMP-AMP synthase (cGAS) and cyclic GMP-AMP (cGAMP), leading to the production of type 1 interferons (IFN-α and IFN-β) (20, 21). To further investigate whether STING signals were enhanced in responders, we calculated STING signature scores (i.e. geometric mean of 44 STING genes’ expression) (22) and compared the change between on-treatment and baseline for each patient. Remarkably, all molecular responders (participants 2, 5, and 11) showed 1.5- to 2-fold increase in STING signature score after the treatment, whereas nonresponders had minimal or even decreased STING gene expression (Figure 5E). This finding suggests an induced viral mimicry immunogenic response among the molecular responders.

### Immune-related gene sets were significantly hypomethylated and upregulated after low-dose 5-aza treatment

**Figure 6:**
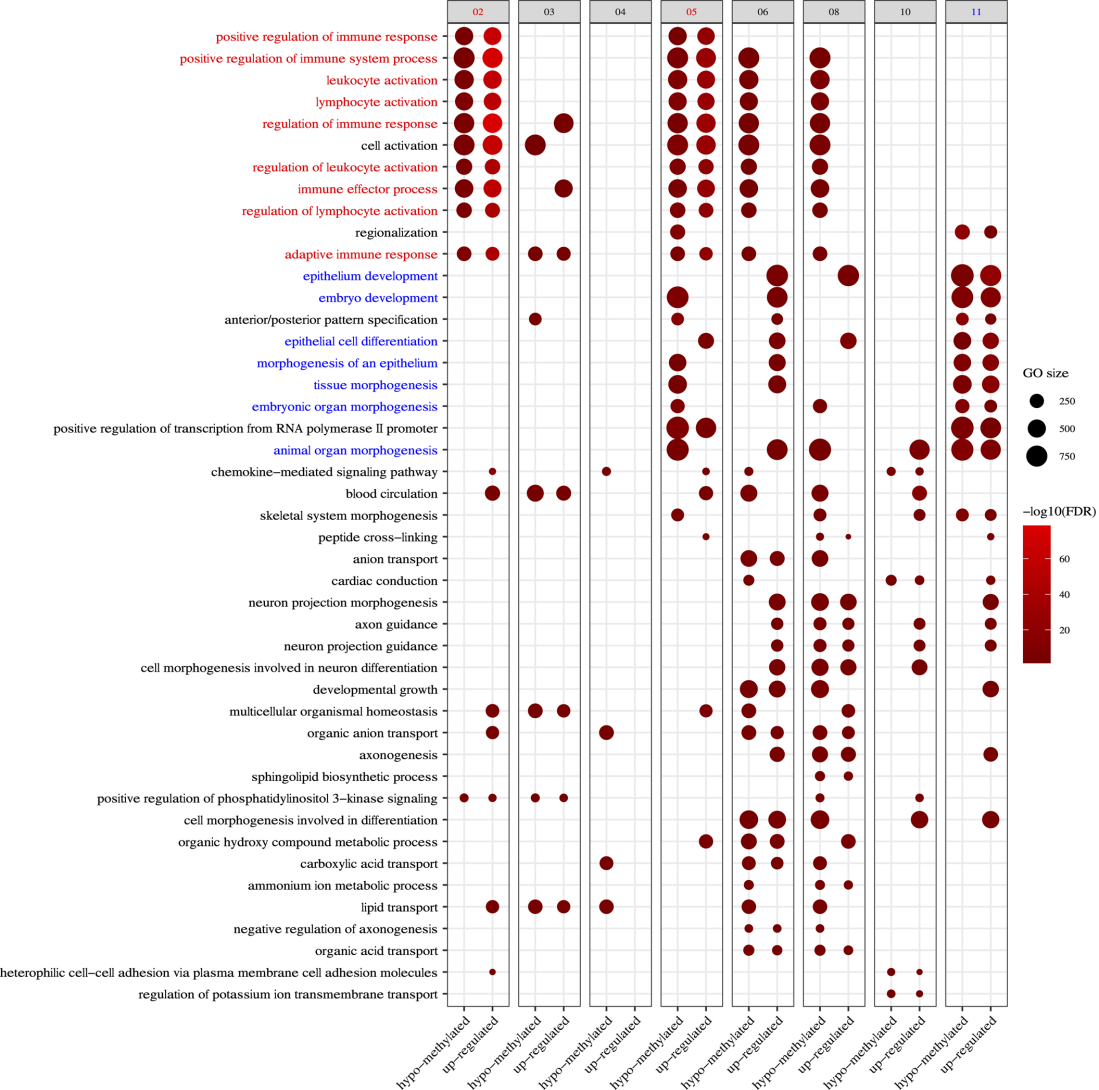
Gene set enrichment analysis of both methylation and expression datasets demonstrate hypomethylation and upregulation of immune response gene pathways (Participants 2 and 5) and developmental and differentiation pathways (Participant 11). Participants 2 and 5 show simultaneous hypomethylation and upregulation of immune activation and response pathways (red), while participant 11 shows simultaneous hypomethylation and upregulation of developmental, differentiation, and morphogenesis pathways (blue). The size of each dot represents the number of genes associated with the GOBP terms, and the intensity scale of the dot represents the –log_10_(*P*).

Using an unbiased approach, we then determined which tumor and/or immune-related pathways were markedly hypomethylated and upregulated in each paired biopsy sample. By visualizing the top 5 statistically significant hypomethylated and upregulated Gene Ontology biological process (GO BP) terms in each participant, we observed that participants 2 and 5 had strong responses in *lymphocyte activation, regulation of immune response*, and related terms (Figure 6 and Supplemental Tables 10 and 11), whereas participant 11 had an overrepresentation of hypomethylation and upregulation in *embryo development*, *epithelial cell differentiation*, *tissue morphogenesis*, *regionalization*, and other related terms, consistent with the observed upregulation of CTA expression. None of the other participants had a notable group of cancer-related terms as significant as the above.

### Hypomethylation and increased expression of tumor suppressor genes observed with low dose 5-aza treatment

**Figure 7:**
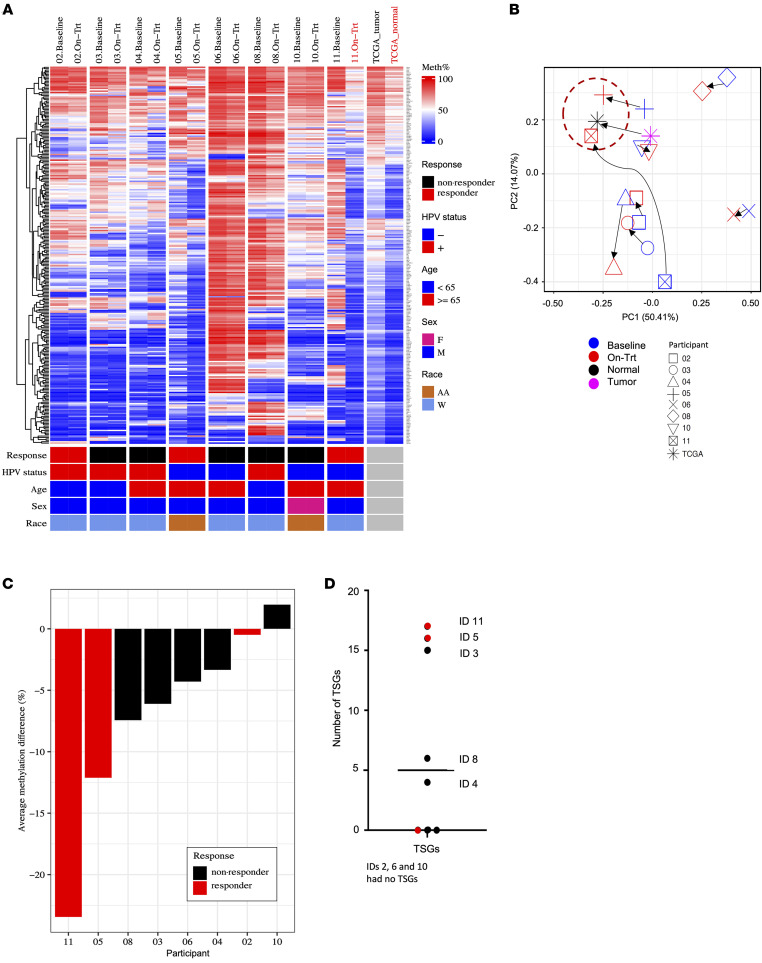
5-aza treatment resulted in hypomethylation of tumor suppressor genes (TSGs). (**A**) Treatment with 5-aza resulted in hypomethylation of TSGs commonly hypermethylated in HNSCCs. Specifically, the methylation profile of Participants 5 and 11 reversed to a methylation pattern observed in noncancer tissue samples representative within the HNSCC cohort in the Tumor Cancer Genome Atlas (TCGA). (**B**) PCA analysis based on the methylation of TSGs demonstrated that the on-treatment samples from participants 5 and 11 were most similar to normal tissue within the TCGA-HNSC cohorts. (**C**) Participants 5 and 11 had the greatest average decrease in methylation, approximately 12% and approximately 23%, respectively. (**D**) Participants 5 and 11 also had the greatest number of TSGs with at least a 25% hypomethylation change and 2-fold increase in expression.

Tumor suppressor genes (TSGs) are known to be hypermethylated in HNSCC (23, 24). Therefore, we sought to investigate whether 5-aza treatment could alter the methylation status of TSGs. We identified 268 TSGs with hypermethylated promoters in HNSCCs between tumor and normal samples (see Methods; Supplemental Table 12), as identified in the TCGA-HNSC methylation array data (13) and assessed the methylation changes in these 268 TSGs in our cohort. The on-treatment methylation profile of 2 participants (participants 5 and 11) most resembled the normal, noncancer samples in the TCGA-HNSC dataset (Figure 7A). Concordantly, the on-treatment samples of these 2 participants clustered with the normal samples in TCGA-HNSC using principal component analysis (PCA) (Figure 7B). These 2 participants also had the greatest decrease in methylation of the TSGs (average methylation decrease for participants 5 and 11 was approximately 12% and approximately 23%, respectively) (Figure 7C). Together, these results suggest low-dose 5-aza could reverse the TSG hypermethylation phenotype to a near-normal methylation signature in participants 5 and 11. Further analysis of the number of TSGs with more than a 25% decrease in promoter methylation and an associated more than 2-fold increase in expression showed that participants 3, 5, and 11 had equal or more than 15 TSGs reexpressed in the tumor after treatment (Figure 7D). Some of the hypomethylated and reexpressed TSGs identified in participants 5 and 11 include important cellular genes relevant to tumorigenesis such as *ST5, IRX1, MAP3K8, PRDM1,* and *SOX7*.

## Discussion

ICB has revolutionized the treatment of patients diagnosed with R/M HNSCC (3, 4, 25–27). However, more than 80% of patients with R/M disease progress on ICB treatment (2) and, at the time of tumor recurrence, OS is 6–12 months (28). Salvage radiotherapy (29) and further adjuvant chemotherapy (5, 30) have not improved outcomes for patients with R/M disease who have progressed on ICB. Thus, we performed an investigator-initiated Phase 1b clinical trial that assessed the effects of a DNA methyltransferase inhibitor, 5-aza, in participants diagnosed with R/M HNSCC. Given that HLA class I APM downregulation or loss is a known mechanism of immune evasion utilized by HNSCC (31), we explored the use of 5-aza to improve the tumor antigenicity of HNSCCs and improve clinical outcomes upon rechallenge with ICB therapy.

Since 5-aza is FDA-approved for treating myelodysplastic syndrome and myelomonocytic leukemia, we were concerned about its potential off-target hematologic effects. Thus, we evaluated 2 regimens of low-dose 5-aza (40 mg/m^2^), a 5- and a 10-day treatment course per cycle to assess whether 5-aza can modulate the TME sufficiently without the associated hematological toxicity. As shown in Supplemental Figure 1, although there was no difference in the global methylation changes of participants between the two dose cohorts, our data suggest that a 5-day course of low dose 5-aza provides a similar capacity for epigenetic reprogramming with fewer hematological adverse effects (Table 3).

Several studies have reported on the association between NLR and clinical efficacy in patients treated with immunotherapy. A large metaanalysis was performed that included 4,154 cancer patients across 38 studies (32). Six studies demonstrated that the NLR level in patients without a clinical response to immunotherapy may increase after immunotherapy treatment. The upward trend in NLR was associated with a shorter OS (pooled HR: 2.05, 95% CI: 1.79–2.35, *P* < 0.001) and a downward trend in NLR was associated with longer OS (pooled OR: 0.49, 95% CI: 0.42–0.58, *P* < 0.001) (32). In our trial, after low-dose 5-aza treatment, a greater-than 50% reduction in mean NLR (14.2 to 6.9) was observed. Moreover, not only was systemic reprogramming of the host immune response detected after 5-aza treatment, but also local reprogramming within the TME identified through the study of paired pre- and on-treatment biopsy samples.

Thirty-eight percent (3 of 8) of participants with evaluable paired tissue samples had a greater than 2-fold increase from baseline in the IFN-γ, *CD274,* and STING signaling pathways within the TME after 5-aza treatment. Our data support findings from prior studies that demonstrate that increased IFN-γ, *CD274,* and STING signaling correlate with response to ICB (2, 7, 33). The increase in STING signaling upon low-dose 5-aza administration suggests STING-induced viral mimicry immunogenicity. However, no noticeable increase in the expression of endogenous retroviruses (ERVs), mitochondrial antiviral signaling gene (*MAVS*), or 6 repetitive elements (Alu, L1, L2, LTR, MIR and Satellite) was observed (data not shown). This indicates that the induction of STING signaling was not likely due to reactivation of those elements. Instead, our results suggest several other mechanisms for the observed increase in biomarkers of response to ICB with 5-aza treatment. One mechanism is through increased HLA and APM expression in the setting of increased expression of CTAs, both of which are associated with increased CD8^+^ T cell infiltration. Another mechanism may be the hypomethylation and reexpression of TSGs, as we observed a 12%–23% decrease in promoter methylation regions of TSGs among responders. The role of TSGs in modulating host antitumor immune responses has not yet been fully explored in the field.

Most importantly, the reprogramming of the TME with low-dose epigenetic therapy and associated increases in biomarker signatures correlated with prolonged OS. From the KEYNOTE-40 trial, the median OS in the intention-to-treat population was 8.4 months (95% CI 6.4–9.4) with pembrolizumab monotherapy in participants diagnosed with R/M HNSCC who had progressed on IC (27). However, prior treatment with 3 or more systemic regimens administered for R/M disease was an exclusion criterion to the clinical trial. In the KEYNOTE-048 trial, pembrolizumab alone or with chemotherapy was administered in the first-line therapy setting. The median OS was 11.6 months (95% CI 10.5–13.6 months) in the pembrolizumab-alone treated group, and the median OS was 13.0 months (95% CI 10.9–14.7 months) in the pembrolizumab-plus-chemotherapy–treated group (4). In a Phase III clinical trial administering anti-PD1 therapy to both HPV positive and negative HNSCC patients (CheckMate 141), a post-hoc exploratory analysis was performed involving 178 patients with R/M HNSCC for whom tumor p16 status was available. Among head and neck cancer patients with p16-positive tumors, the median OS was 9.1 months after treatment with anti-PD1 therapy. Among patients with p16-negative tumors, the median OS was 7.5 months (3).

In our clinical trial, 42% (5 of 12) of the treated participants with R/M HNSCC had HPV-mediated HNSCC, which is a population with known improved OS. Furthermore, as part of the inclusion criteria, all participants had progressed beyond several lines of treatment, including ICB therapy. Across the treated participants in our trial, the median OS was 16.3 months, a 2-year OS rate was 24.7% (95% CI: 4.5%, 53.2%), and 58% of participants had greater than 12-month OS after the initiation of study treatment, suggesting that 5-aza in combination with ICB has a clinically meaningful impact independent of HPV status. Our trial was terminated early due to the hospital constraints of running clinical trials and performing tumor biopsies during the COVID-19 pandemic. However, the available data from the treated participants highlight several important concepts. First, in the era of combinatorial immunotherapeutic strategies, the incorporation of biomarker analyses can assist in determining treatment efficacy, especially in small cohort studies. Second, intervening therapies can reprogram the TME and alter PD-L1 levels (34). Therefore, retesting for PD-L1 expression between intervening therapies may help to provide a rationale for rechallenge with ICB. We conclude that low-dose 5-aza can reprogram systemic and local host immune responses sufficiently to increase the expression of molecular markers of response to ICB, and that tumor and/or immune cell reprogramming may translate into prolonged OS compared with historical controls.

## Methods

### Sex as a biological variable.

Our study was open to enrollment to both male and female participants. HNSCC has an estimated 3:1 higher incidence in men than in women (35), and an even higher imbalance in HPV-mediated HNSCCs, wherein approximately 80% of HPV-mediated HNSCC cases are in men in the United States (U.S.) (36). The distribution of our clinical trial cohort reflects the sex prevalence of HNSCC in the U.S.

### Study participants.

The study was a clinical trial performed at the Massachusetts General Hospital (Boston, MA, USA). Participants were eligible for enrollment if they were diagnosed with R/M HNSCC and had progressed on ICB therapy (anti-PD-1, anti-PD-L1, anti-cytotoxic T lymphocyte associated protein 4 (CTLA4)) (NCT03019003). Other inclusion criteria were the presence of measurable disease according to RECIST 1.1, and an Eastern Cooperative Oncology Group (ECOG) performance status of 0 or 1.

### Therapy timing and dosing.

Participants received low dose 5-aza (40 mg/m^2^) via subcutaneous (s.c.) injection either on days 1–5 (‘dose level 1,’ 5-day treatment group), or low dose 5-aza (40 mg/m^2^) on days 1–5 and 8–12 (‘dose level 2,’ 10-day treatment group) of each 4-week cycle for up to 12 cycles. All participants received durvalumab 1,500 mg intravenously on day 1 of each 4-week cycle for up to 12 cycles and tremelimumab 75 mg intravenously on day 1 of each 4-week cycle for up to 4 cycles. 5-aza was administered alone in cycle 1 to prime the tumors and the combination of 5-aza, durvalumab, and tremelimumab therapy started in cycle 2. An amendment was added in 2019 to replace the s.c. injection with oral decitabine and to remove tremelimumab.

### Trial objectives and disease assessment.

The primary objective was to determine the BED of 5-aza after s.c. injection in combination with durvalumab and tremelimumab. BED was defined as the dose of the DNA methyltransferase inhibitor that resulted in at least a 10% decrease in molecular markers from the baseline biopsy to the 8-week on-treatment tumor biopsy in at least 50% of participants within the same dose level. Two dose levels were tested.

The second objective was safety, and each dose was also assessed for safety. Adverse events (i.e. dose limiting toxicity [DLT]) were to be assessed through 28 days after cycle 2. A total of 6 participants were to be entered into dose level 1. If less than 2 of the 6 participants treated at dose level 1 experienced a DLT, a subsequent group of 6 participants were to receive dose level 2. If 2 or more of the first 6 subjects experienced a DLT, then the dose was to be reduced, and an additional 6 participants were to be treated at the reduced dose. If 2 or more of the 6 participants at the reduced dose experienced a DLT, then accrual to the study was to be suspended. Approximately 22 participants were estimated to be accrued (*n* = 6 across 2 doses plus *n* = 10 into an expansion cohort).

### Sample collection.

Per protocol, a paired biopsy and peripheral blood draw at baseline (pretreatment) and at week 8 (on treatment) were to be performed.

### NLR in the peripheral blood.

NLR was calculated by dividing the absolute number of neutrophils by the absolute number of lymphocytes.

### Tissue sample preparation for methylation EPIC Array and RNA-seq.

Core tissue biopsies were performed under image guidance to ensure the biopsy was targeted to the tumor. H&E-stained slides were reviewed by in-house pathologists to confirm presence of tumor. DNA and RNA coextractions were performed by the Broad Institute of MIT and Harvard using Qiagen AllPrep DNA/RNA kits (Cat. 80204). RNA samples were quantified using Qubit 2.0 Fluorometer and RNA integrity was analyzed using an Agilent TapeStation. Genome-wide DNA methylation was assessed using the Illumina Infinium Methylati1PIC BeadChip array that provides a genome-scale interrogation of the entire human methylome, with the sampling of more-than 850,000 CpG sites in the human genome by the USC Molecular Genomics Core. RNA library preparations, sequencing reactions, and initial bioinformatics analyses were conducted at GENEWIZ, LLC.

### Genome-wide DNA methylation analysis.

Raw data files of Infinium MethylationEPIC BeadChip were quality controlled, preprocessed, and normalized using the *ChAMP* Bioconductor package (v2.16.2) in R (v3.6.0) (37). The Slide and Plate ID were identified to be substantial batch effects by *champ.SVD* function, and the batch effects were corrected by *champ.runCombat*. The CpG sites exhibiting high detection *P* value (detection *P* > 0.01) or low bead count (< 3) for over 5% of samples were excluded. Probes with SNPs, that aligned to multiple locations, and non-CpG probes or probes from X and Y chromosomes were also excluded. In total, 674,322 probes were retained for downstream analysis. A β value, defined as the ratio between methylated (M) and total signal intensities (M + unmethylated [U]) ranging from 0 (fully unmethylated) to 1 (fully methylated), was assigned to each CpG site, and its log_2_ transformed M value (i.e. log_2_(β/1 – β)) was used in the differential probe/region analysis. The differentially methylated probe (DMP) and differentially methylated region (DMR) analysis between the paired on-treatment and baseline samples was conducted using the *champ.PairedDMP* and *champ.PairedDMR* functions. The *bumphunter* (38) method was used to tile the CpG sites with default settings. Significant DMPs were identified using Benjamini-Hochberg adjusted *P* value < 0.05, and the significant DMRs using the permutation-adjusted *p.valueArea* cutoff < 0.05. The Methylati1PIC BeadChip data is available at the European Genome-Phenome Archive as described below.

### RNA-seq analysis.

The raw RNA-seq data were aligned to hg38 using STAR 2.7.1a (39) with default alignment parameters. Quality control was performed using FastQC and MultiQC (40) before and after alignment. Gene expression levels were quantified using HTSeq v0.11.2 with the “union” mode option and alignment quality > 30 (41). Normalization and calculation of log_2_RPKM values (log_2_ Reads Per Kilobase of transcript, per Million mapped reads) were performed using the *rpkm* function in the edgeR Bioconductor package (42). The batch effect was corrected using the *RUVg* method in the RUVSeq R Bioconductor package (43). Differentially expressed genes (DEG) between the paired on-treatment and baseline samples were identified using the model with the group (baseline, 5-day treatment group (dose level 1), and 10-day treatment group (dose level 2)) as the main factor and participant as a covariate. The *glmQLFTest* was used to identify significant differential genes (FDR < 0.05) for on-treatment-5 day versus baseline or on-treatment-10 day vs. baseline comparisons. The RNA-seq data is available at European Genome-Phenome Archive as described below.

### Gene lists used to assess tumor antigenicity and regulation.

The 6 genes associated with IFN-γ signaling were reported by Ayers et al. (7), and the geometric means of the expression of the 6 genes(RPKM) were calculated and defined to be the IFN-γ signature scores for each sample. The genes related to APM (*n* = 15, Supplemental Table 8) and cancer testis genes (*n* = 1,019, Supplemental Table 9) were extracted from Andtbacka et al. and Almeida et al., respectively (44, 45). Tumor suppressor genes (TSGs) (*n* = 1,217, Supplemental Table 12 were downloaded from the Tumor Suppressor Gene Database (https://bioinfo.uth.edu/TSGene/download.cgi?csrt=7565779572534791767) on February 3, 2023.

To further identify hypermethylated TSGs in HNSCC, we first downloaded Illumina’s Infinium HumanMethylation450 BeadChip data for the TCGA-HNSC cohort (https://www.cancer.gov/tcga), including 528 HNSCC and 50 normal samples. The eBayes approach implemented in the R package *limma* (46) was employed to identify the differentially methylated probes (DMPs) between tumor and normal based on the cutoffs of FDR < 0.05 and absolute methylation difference ≥ 10%. The genomic locations of the CpG probes were annotated by Illumina’s accompanying annotation data file (IlluminaHumanMethylation450kanno.ilmn12.hg19 (47)). Next, we extracted the subset of the downloaded TSGs with at least 1 hypermethylated promoter DMPs (hyper-DMPs, methylation difference ≥ 10%) between tumor and normal in TCGA-HNSC and examined their methylation levels in our cohort (Supplemental Table 12).

### Cell type deconvolution using DNA methylation data.

MethylCIBERSORT was applied on the Methylati1pic BeadChip data to estimate 10 cell type fractions in each sample (48): Fibroblasts, Neutrophils, CD4^+^ T, CD8^+^ T, Treg, CD14 [Macrophages], CD19 [B cells], CD56 [NK cells], Eosinophils, and Cancer cells. The reference for cell type deconvolution, intrinsic to MethylCIBERSORT, and specifically designed for Head and Neck cancer, contained 1,197 CpG sites. We intersected the reference with our data to retain only the overlapping CpG sites, resulting in 933 CpGs, and proceeded with the analysis via the online platform (https://cibersort.stanford.edu).

### IHC and scoring.

Five-micron–thick unstained tissue slides from formalin-fixed, paraffin-embedded (FFPE) tissue were cut onto charged slides. For IHC, slide pretreatment and staining used reagents provided with the EnVision Flex Mini Kit, High pH (Agilent Dako, Cat K8023) and followed the manufacturer’s recommendations. In brief, antigen retrieval (pH 9) was performed using a pressure cooker for 2 minutes at 120° C followed by stepwise cooling. Slides were incubated overnight with primary antibodies against CD8 (CD8-4B11-L-CE Thermo Fisher Scientific) and FOXP3 (Ab20034 Abcam) at 4° C at a 1:400 and 1:100 dilution, respectively. After washing, slides were incubated with Mouse Linker (K802221 Dako), and slides were developed using a di-amino-benzidine-peroxide (DAB) substrate kit (SK-4100 Vector Labs) for 1 minute and 90 seconds, respectively. Slides were counterstained with hematoxylin. The CD8 and FOXP3 antibodies were previously validated and titrated on normal tonsil sections, and antibody specificity and staining quality were assured by in-house board certified head and neck pathologists (49). A semiquantitative evaluation of the membrane CD8 and nuclear FOXP3 IHC staining intensity (rated as follows: 0, no staining; 1, weak; 2, median; or 3, strong) and the proportion of positive cells by a pathologist were used to generate the total Histoscore (H-score) according to the formula: 1 × (% cells with staining intensity 1) + 2 × (% cells with staining intensity 2) + 3 × (% cells with staining intensity 3), generating a total score ranging from 0 to 300.

### GSE testing.

GSE analyses were performed on both transcriptional and methylation data. The differential gene expression between on treatment vs. baseline for each participant was analyzed by *edgeR exactTest*, and the genes were ranked by *P* values, followed by GSE testing using directional *LRpath* (50) and gene sets from the Gene Ontology Biological Processes (GO BP) database. For each participant, the methylation differences in β values of CpG sites at the promoter regions (TSS200 or 5’UTR) were calculated between on-treatment and baseline samples, then standardized to Z-scores. The gene-level Z-scores were combined Z-scores of CpGs annotated to the same gene divided by the square root of the number of CpG sites:

 (Equation 1)
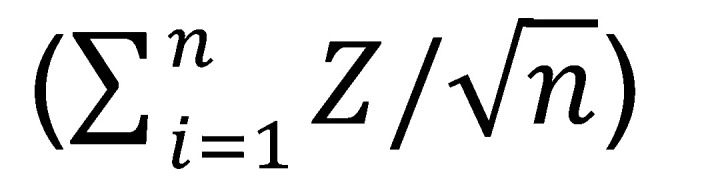


Finally, the *P* values of genes were determined based on the Z-scores. Using the calculated *P* values, *LRpath* (http://lrpath-db.med.umich.edu) (50, 51) was applied to test enrichment of GO BP gene sets. Significant GO BP terms (FDR < 0.05) for both gene expression and methylation were selected. GO BP terms with both significant hypomethylation and upregulated gene expression in the on-treatment sample compared with baseline were of particular interest and used for visualization.

### Statistics.

Radiographic assessment was performed every 8 weeks from cycle 1 day 1. Participants were followed up to 2 years from study registration. The primary analysis included eligible participants who at least began protocol treatment. The Kaplan-Meier method, with corresponding 95% confidence intervals was used to estimate time-to-event distributions. Correlative biomarker analyses were reported among those with an evaluable pre- and on-treatment (8-week) biopsy sample available and analyzed using paired 2-tailed *t* tests. Wilcoxon rank-sum test was used for 2-group comparisons. Two-sided *P*-values were reported, and *P* < 0.05 was considered statistically significant. Statistical analysis was performed in R (version 4.3.2).

### Study approval.

The study protocol was approved by the Institutional Review Board of Dana-Farber Harvard Cancer Center (DFHCC#16-425) and was registered with the National Institutes of Health (NIH) U.S. National Library of Medicine clinicaltrials.gov website (NCT03019003) and conducted in accordance with the Declaration of Helsinki. The U.S. Food and Drug Administration also reviewed and approved the protocol under Investigational New Drug Application #133011. The trial had oversight from the DFHCC Data and Safety Monitoring Committee (DSMC). Written informed consent was obtained from all the participants prior to enrollment. All participant numbers presented in the text and figures are deidentified identification numbers (IDs).

### Data availability.

Values for all data points are available in the Supplemental Data Values file and from the corresponding author upon request. The sequencing data was deposited at European Genome-Phenome Archive, and the accession number is EGA50000000682.

## Author contributions

SIP developed the concept. SIP, JCP, RH, KYS, and AO designed the clinical trial. TQ, AKM, JSC, KYS, SL, GM, AJD, CSG, MJP, TRM, AO, SL, MAS, and SIP analyzed, interpreted the experimental data, organized the figures, and contributed to the initial writing of the manuscript. MJP, TRM, and SIP were responsible for obtaining funding for the study. PMS and WCF reviewed H&E-stained slides to confirm the presence of tumor. GM and AJD performed the IHC staining. All authors reviewed and edited the final manuscript and provided critical feedback. For cofirst authors, the order of authorship was assigned based on contribution to the data analysis.

## Supplementary Material

Supplemental data

ICMJE disclosure forms

Supplemental table 1

Supplemental table 10

Supplemental table 11

Supplemental table 12

Supplemental table 2

Supplemental table 3

Supplemental table 4

Supplemental table 5

Supplemental table 6

Supplemental table 7

Supplemental table 8

Supplemental table 9

Supporting data values

## Figures and Tables

**Table 3:**
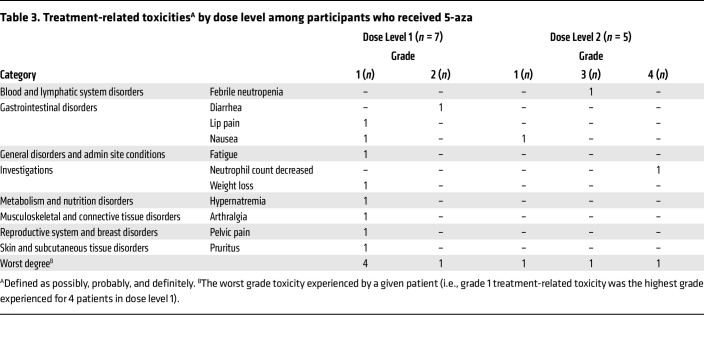
Treatment-related toxicities^A^ by dose level among participants who received 5-aza

**Table 2:**
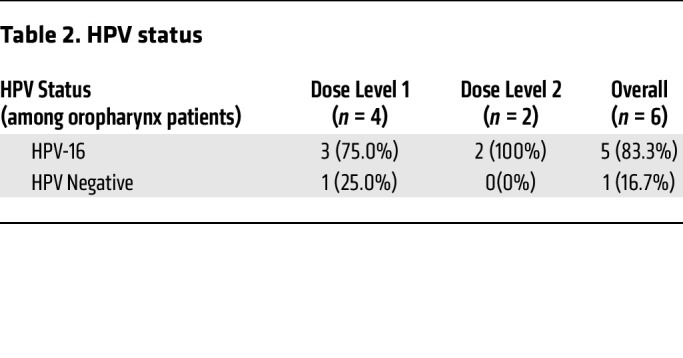
HPV status

**Table 1:**
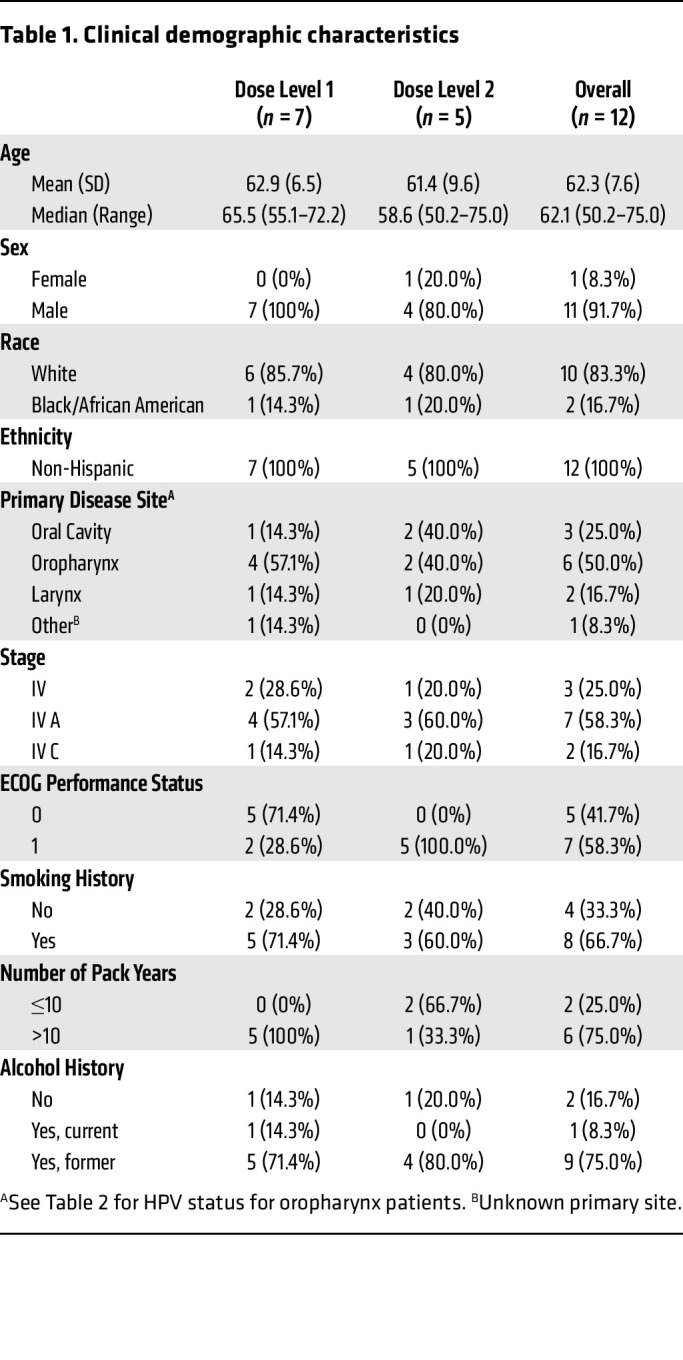
Clinical demographic characteristics

## Version 1 03/17/2025

Electronic publication
