# Supplementary figures and images for: Epigenetic therapy sensitizes anti–PD-1 refractory head and neck cancers to immunotherapy rechallenge
Source: J Clin Invest. 2025 Mar 17;135(6):e181671. doi: 10.1172/JCI181671 (PMC11910227; doi:10.1172/JCI181671)

Figure S1

A

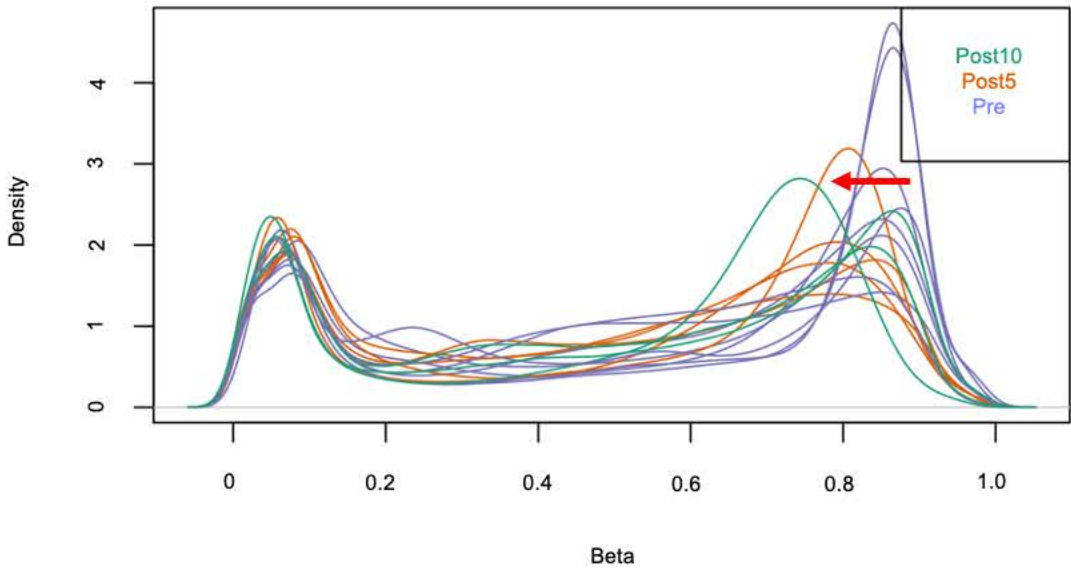

B

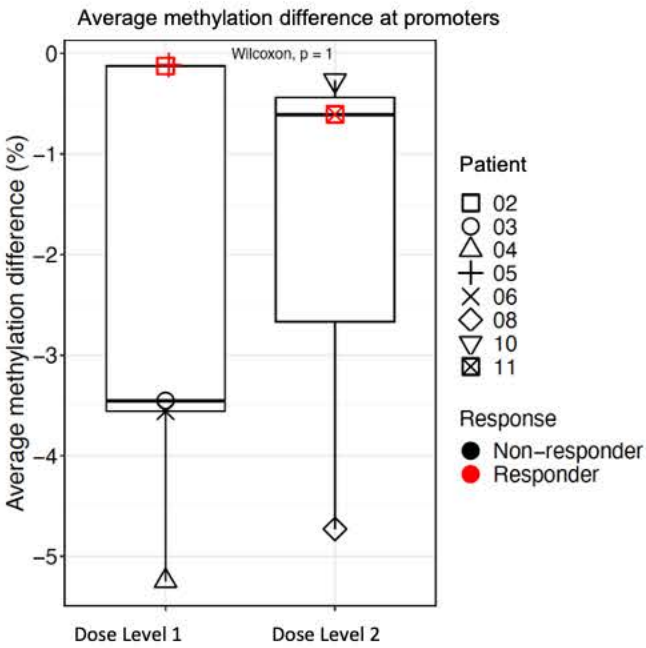

C

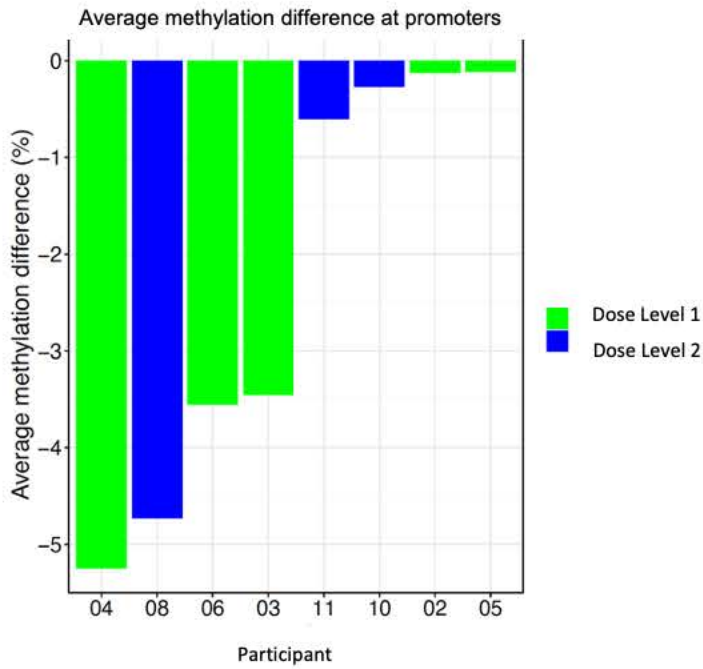

D

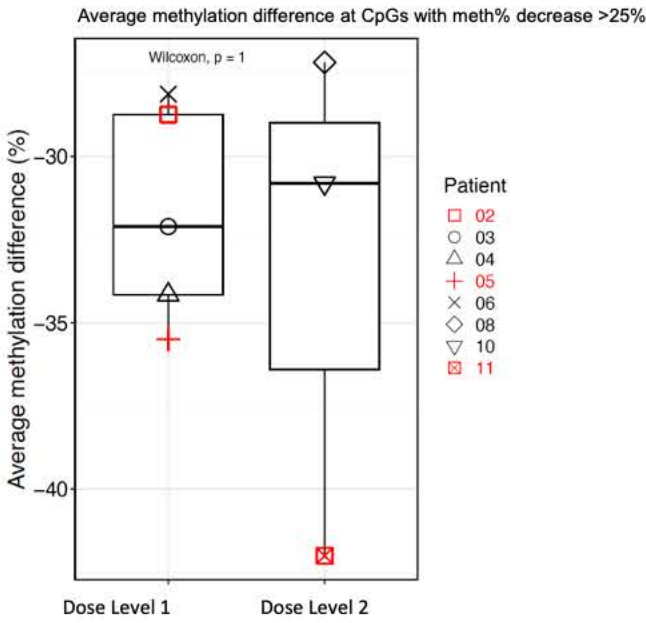

E

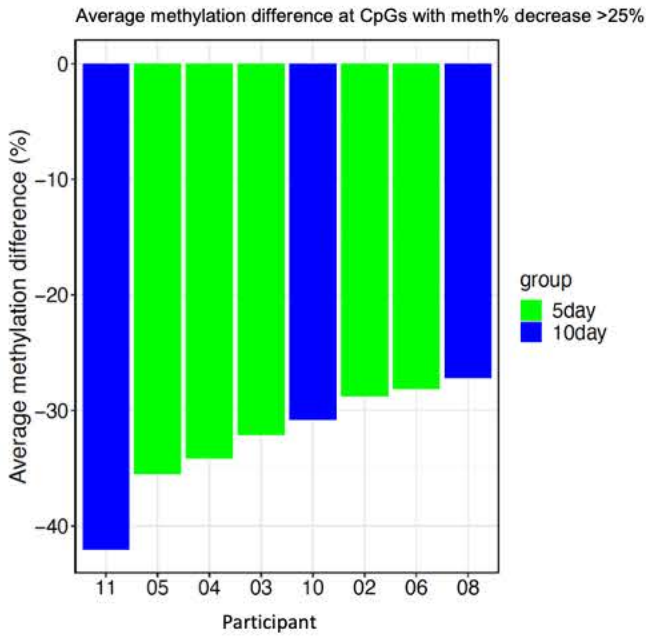

F

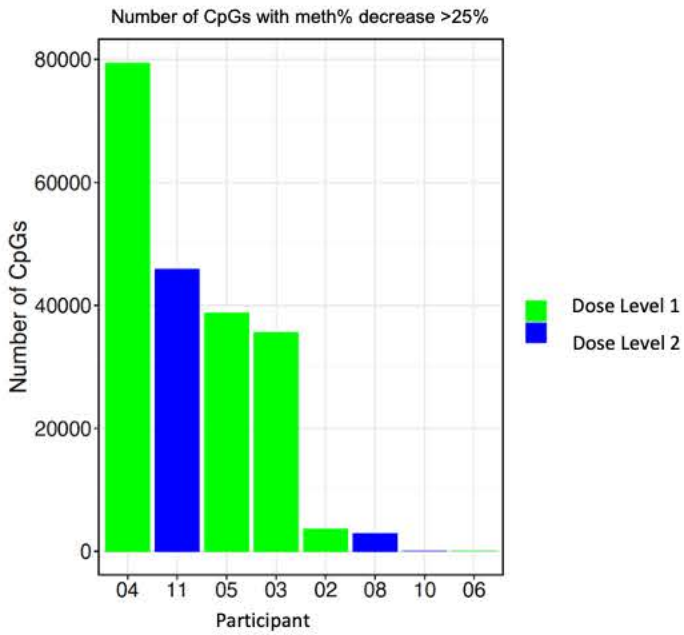

Figure S2

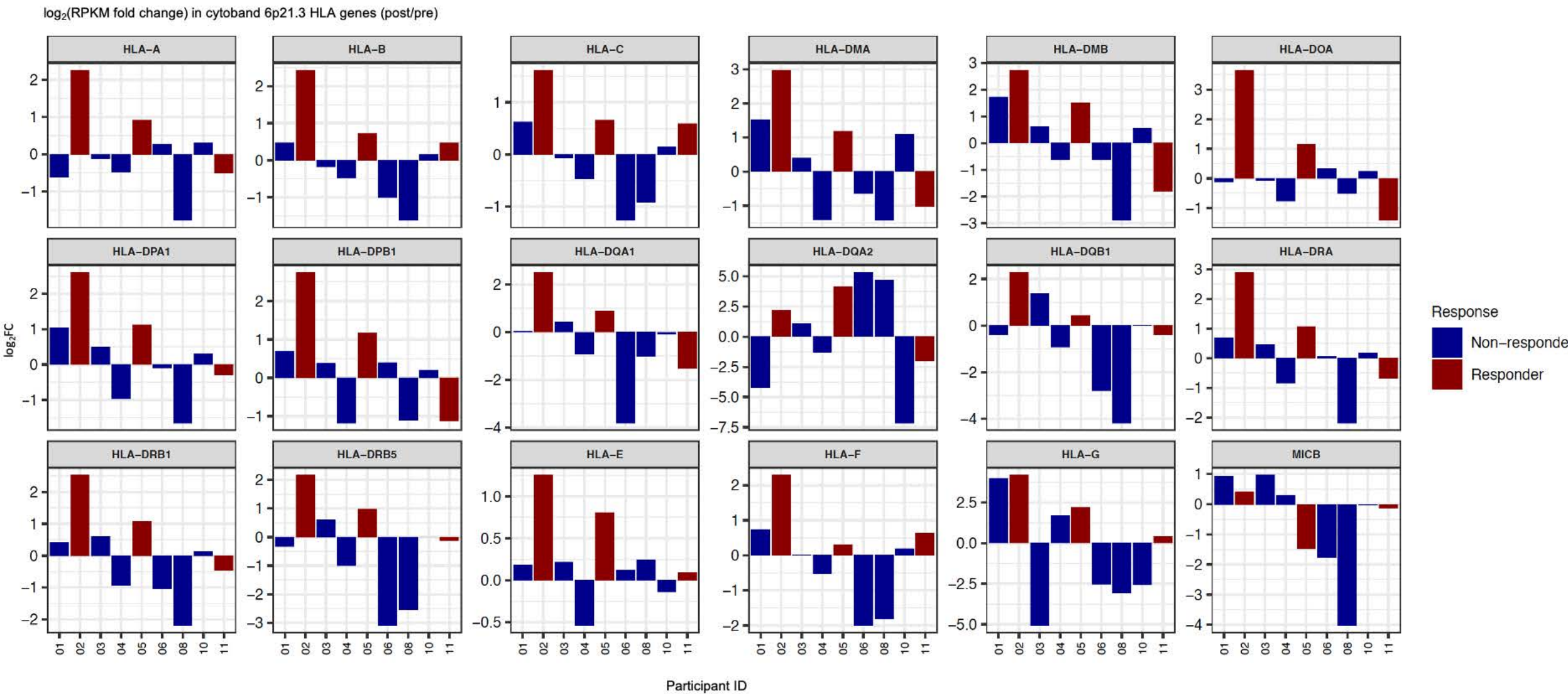

Supplement: Supplemental data [file jci-135-181671-s212.pdf]
